# Tyrosinase Nanoparticles: Understanding the Melanogenesis Pathway by Isolating the Products of Tyrosinase Enzymatic Reaction

**DOI:** 10.3390/ijms22020734

**Published:** 2021-01-13

**Authors:** Paul K. Varghese, Mones Abu-Asab, Emilios K. Dimitriadis, Monika B. Dolinska, George P. Morcos, Yuri V. Sergeev

**Affiliations:** 1 National Eye Institute, National Institutes of Health, Bethesda, MD 20892, USA; paul.varghese@nih.gov (P.K.V.); mones@mail.nih.gov (M.A.-A.); dolinskam@nei.nih.gov (M.B.D.); gpm7rv@virginia.edu (G.P.M.); 2 NIH Shared Resources on Biomedical Engineering and Physical Science, National Institute of Biomedical Imaging and Bioengineering, National Institutes of Health, Bethesda, MD 20892, USA; dimitre@helix.nih.gov

**Keywords:** oculocutaneous albinism, protein purification, tyrosinase, tyrosinase nanoparticles, dopachrome/melanin purification

## Abstract

Human Tyrosinase (Tyr) is the rate-limiting enzyme of the melanogenesis pathway. Tyr catalyzes the oxidation of the substrate L-DOPA into dopachrome and melanin. Currently, the characterization of dopachrome-related products is difficult due to the absence of a simple way to partition dopachrome from protein fraction. Here, we immobilize catalytically pure recombinant human Tyr domain (residues 19–469) containing 6xHis tag to Ni-loaded magnetic beads (MB). Transmission electron microscopy revealed Tyr-MB were within limits of 168.2 ± 24.4 nm while the dark-brown melanin images showed single and polymerized melanin with a diameter of 121.4 ± 18.1 nm. Using Hill kinetics, we show that Tyr-MB has a catalytic activity similar to that of intact Tyr. The diphenol oxidase reactions of L-DOPA show an increase of dopachrome formation with the number of MB and with temperature. At 50 °C, Tyr-MB shows some residual catalytic activity suggesting that the immobilized Tyr has increased protein stability. In contrast, under 37 °C, the dopachrome product, which is isolated from Tyr-MB particles, shows that dopachrome has an orange-brown color that is different from the color of the mixture of L-DOPA, Tyr, and dopachrome. In the future, Tyr-MB could be used for large-scale productions of dopachrome and melanin-related products and finding a treatment for oculocutaneous albinism-inherited diseases.

## 1. Introduction

The melanogenesis pathway describes an intricate process that results in pigmentation in the skin, eyes, and hair. This pathway takes place within the melanosomes of melanocytes where pigmentation is a result of the production and packing of a biopolymer, melanin. A reduction of melanin formation due to point mutations of the enzymes involved in the melanogenesis pathway, leading to a series of autosomal recessive disorders, has been studied and is classified under oculocutaneous albinism (OCA). Although variable between geographic locations, the estimated prevalence of all types of oculocutaneous albinism worldwide is 1:30–40,000 with an average of 1:17,000 people being affected [1]. Most well-known of the enzymes involved in the melanogenesis pathway is human tyrosinase (Tyr) where genetic mutations can be found in the *TYR* gene. Specifically, OCA1 consists of a bi-allelic mutation in this *TYR* gene and happens to be the most common type of albinism. Although a diagnosis is formulated by clinical findings of common ocular symptoms and hypopigmentation, these symptoms often overlap, so establishing the genetic causal effects has proved to be important. The clinical spectrum for degree of severity in OCA1 ranges between OCA1A as the most severe type, causing a complete loss of melanin production due to a loss of Tyr activity, and the less severe OCA1B, which is marked by reduced Tyr activity in melanin production. These two subtypes of OCA1 cause significant changes phenotypically ranging from a loss or reduction of pigmentation, reduced visual acuity, and photophobia to nystagmus, strabismus, and reduced stereoscopic vision [2]. It has been proposed that OCA1 is a result of endoplasmic reticulum (ER) retention disease where misfolded mutant Tyr are retained in the ER and unable to attain complex glycan structures from the cis and trans-Golgi apparatus [3]. Although there is no effective treatment for OCA currently, chemical chaperone therapy, which uses competitive inhibitors to achieve partial catalytic activity of Tyr, has provided a possible route for investigation, but the effects are variable as it assists in the folding of some mutant Tyr but not others [4]. Many *TYR* gene mutations (more than 350 in all) affect the activity of Tyr either fully or partially resulting in an alteration of eumelanin production.

The rate limiting enzyme of the melanogenesis pathway, Tyr, is a 529 amino acid type 1 membrane glycoprotein that consists of an N-terminal signal peptide sequence, EGF-like and tyrosinase domains, and C-terminal transmembrane alpha-helix domain [5]. Tyr catalyzes the hydroxylation of L-tyrosine into L-3,4-dihydroxyphenylalanine (L-DOPA), which is then oxidized to L-dopaquinone and later dopachrome [6]. The transmembrane helix spans the lipid membrane of the melanosome within the melanocyte. Tyr undergoes post-translational modifications in the Golgi apparatus through the addition of asparagine-linked (N-linked) glycans (later modified further with complex sugars) and contains seven N-linked glycosylation sites [7]. These modifications are essential for the maturation, functionality, and stability of Tyr. The active site of Tyr involved in the hydroxylation and oxidation of its substrates contains two copper binding sites, CuA and CuB, which are critical for its catalytic activity and are cooperative [8]. Three histidine residues coordinate with each copper ion. Specifically, H180, H202, and H211 at CuA and H363, H367, and H390 at CuB.

The intra-melanosomal domain of human tyrosinase and mutant variants were recently purified from *Ti. ni*. larval biomass [9]. Two OCA1A and four OCA1B mutant variants were characterized by protein activity, tryptophan fluorescence, and Gibbs-free energy changes to demonstrate a link between mutations, tyrosinase conformational stability, and enzymatic activity [10]. Results of this work suggest an association between tyrosinase protein stability and loss of pigmentation in OCA1B. Membrane-associated human tyrosinase was purified by Kus et al. [11]. The full-length protein demonstrated a similar catalytic activity compared to that of the intra-melanosomal tyrosinase domain. Recently, we demonstrated, using van’t Hoff temperature-dependent analysis and Michaelis-Menten kinetics, that the association of L-DOPA with human tyrosinase is a spontaneous enthalpy-driven reaction, which is unfavorable at the final step of dopachrome formation [5].

The products of the Raper-Mason pathway using tyrosine and L-DOPA as precursors for eumelanogenesis include dopaquinone, dopachrome, 5,6-dihrdoxyindole-2-carboxylic acid (DHICA), and eumelanin [12]. As discussed, both precursors are oxidized to form dopaquinone in the presence of Tyr and then undergo cyclization to form dopachrome [12]. Next, dopachrome goes under spontaneous rearrangement to form DHICA in the presence of dopachrome tautomerase, tyrosinase-related protein 2 (Tyrp 2) [13]. DHICA oxidase, tyrosinase-related protein 1 (Tyrp 1) finally catalyzes the oxidation of DHICA to produce a brown-black eumelanin [13,14]. While melanin provides a protective role for many species including humans, by protecting DNA from UV damage, the process of melanogenesis produces reactive oxygen species and semi-quinone radicals that are toxic to melanocytes [15]. The toxicity of dopachrome is still a subject of inquiry and studies are underway to characterize dopachrome further.

As discussed, dopachrome is a product of the Tyr-enzymatic reaction in the Raper-Mason pathway of melanogenesis. Although unstable due to its ability for self-oxidation, dopachrome is a compound of interest as its production can be used to test for the activity of Tyr while examining its relation to other critical enzymes (Tyrp1 and Tyrp2) in the melanogenesis pathway. Using traditional methods, we previously characterized the enzymatic function of Tyr using the L-DOPA substrate to produce dopachrome and defined its kinetic properties with the presence of protein in solution [5]. To precisely define parameters of dopachrome reaction within the melanogenesis pathway, the issue of dopachrome isolation from Tyr after enzymatic reaction must be resolved to collect more accurate measurements, which has not been achieved before. Traditionally, tyrosinase is incubated together with a substrate to perform a reaction. The result of this reaction is a mixture of tyrosinase, and dopachrome-like products described above. During the incubation with tyrosinase, all small dopachrome-like molecules are formed in a native state. Any further separation, for example, chromatography, of tyrosinase from dopachrome-like products could modify the native state of small molecules. Using tyrosinase magnetic beads does not change the dopachrome native state. In addition, this procedure allows dopachrome and melanin production in a tube on a large-scale. To perform this task, we created a protocol based on Tyr-magnetic bead nanoparticle complexes (Tyr-MB). After performing the tyrosinase reaction, Tyr-MB can be easily removed from the reaction, and the products of the reaction can be scaled up and characterized. Achieving dopachrome isolation would allow for a quantitative characterization of dopachrome and dopachrome-related products. These measurements would further help understand the function of the Tyr active site and examine the role of genetic mutations in OCA1 mutants. In addition, the applicability of isolated dopachrome extends to finding a treatment for pigmentation rescue in OCA1 patients.

In this paper, we suggest an efficient method to isolate the products of the tyrosinase reaction including dopachrome and have performed experiments in quantifying the thermodynamic parameters behind dopachrome production. This was performed by utilizing magnetic nanoparticles at different volumes, executing temperature-dependent experiments at four constant temperatures, and performing dose-dependent experiments. Our in vitro quantitative characterization of melanin-related product and dopachrome by the Tyr enzymatic reaction will illuminate the significance of dopachrome within the melanogenesis pathway and the activity of OCA1 mutant variants. Furthermore, examining these aspects is critical for the potential treatment and foundational insight of the mechanical and molecular basis of OCA.

## 2. Results

### 2.1. Tyr-MB Nanoparticles

Recombinant Tyr was purified with Immobilized Metal Affinity Chromatography (IMAC) and was followed with Size Exclusion Chromatography (SEC) using an ÄKTA Pure chromatography system, as described in the Materials and Methods section. Identity and purity of Tyr were monitored through SDS-PAGE and Western blotting analyses. A colorimetric reaction using the substrate L-DOPA was used to locate which fractions eluted from the chromatography columns contained Tyr. Supplementary Figure S1 shows the ÄKTA Pure chromatography profile for a pure Tyr, SDS-PAGE, Western Blot, Tyr activity, and purity evaluation. Finally, Tyr purification showed a single peak at ~52 kDa suggesting a highly pure protein.

After protein purification, the enzymatic activity of Tyr was assessed by measuring diphenol oxidase activity [9]. The activity was quantified spectrophotometrically at 475 nm using a SpectraMax i3 device by adding a 3 mM L-DOPA solution at a 1:1 ratio with Tyr (2.2 mg/mL) in each well of an activity test plate. The Tyr reaction was visualized by the production of an orange-brown dopachrome formed in each well corresponding to the fractions containing active Tyr. Supplementary Figure S1 (Panel A, Insert a) provides the activity test results of dopachrome production. 

Tyr-MB nanoparticles were prepared using the active Tyr, according to the protocol from the Materials and Methods section. The Tyr was dialyzed, concentrated to 2.2 mg/mL, and immobilized to MB. The active Tyr-MB particles were then incubated with L-DOPA at 37 °C for 30 min. This incubation resulted in the production of dopachrome and dopachrome-like products. Finally, Tyr-MB particles were removed using a magnetic separator to isolate the dopachrome product. Under optimal conditions of 37 °C, the dopachrome product, which is isolated from Tyr-MB particles, shows that dopachrome has an orange-brown color that is different from the color of the mixture of L-DOPA, Tyr, and dopachrome as presented in Supplementary Figure S2. The color was different from the control obtained in the presence of Tyr in the reaction where the color of dopachrome was an opaque and dark-colored mixture.

### 2.2. Tyr-MB: Dose-Dependent Activity 

**Figure 1:**
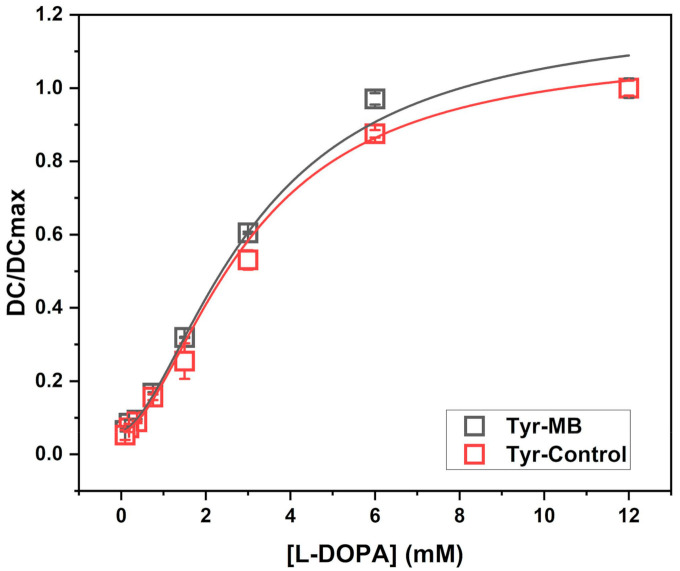
Dopachrome activity of Tyr-MB and Tyr control is described by the dose-response curve. The experimental points were fitted with the Hill equation with the parameters as presented in Table 1 and the caption. For comparison, dopachrome activity, DC, is normalized to the maximum response in dopachrome production, DCmax. Concentrations of L-DOPA substrate ranged with 12, 6, 3, 1.5, 0.75, 0.375, 0.1875, and 0.09375 mM. All measurements were repeated in duplicate and executed independently.

To further establish the enzymatic activity of the Tyr-MB complex, a dose-dependency experiment was performed, and the parameters of Hill equation were calculated for both Tyr control and Tyr-MB. Figure 1 shows that the Hill model describes both the Tyr-MB and the control with high statistical reliability (Table 1). For both samples, the Hill coefficient is *n* > 1. This value indicates that processes of L-DOPA binding and dopachrome formation are positively cooperative for Tyr-MB and the Tyr control. Figure 1 indicates slightly higher dopachrome production within L-DOPA concentrations of 0–6 mM. This observation is confirmed by the value of L-DOPA50, which is the L-DOPA concentration that produces 50% of maximal dopachrome production. This value was 3.18 mM for Tyr-MB. For the Tyr control, a 50% response was achieved at a concentration of 3.01 mM.

### 2.3. Tyr-MB: Dopachrome Production

**Figure 2:**
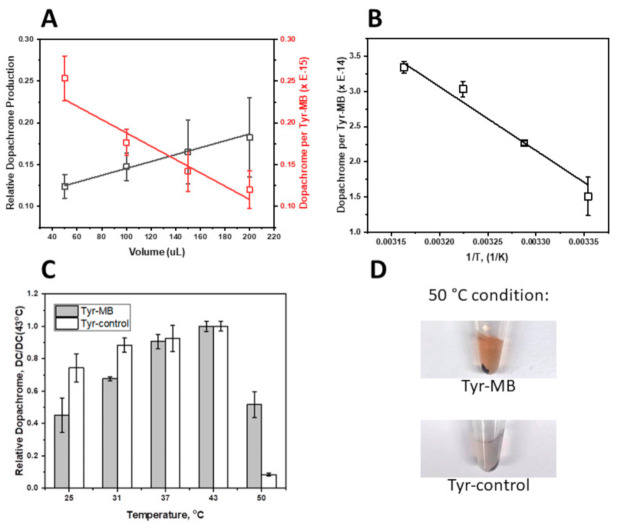
Relative dopachrome production and dopachrome formation per Tyr-MB complex. (**A**) displays the magnetic bead volume-dependent experiment. Since higher volumes of MBs were used, the relative dopachrome production increased (black line, R^2^ = 0.98). This measurement was attained by utilizing a ratio of the relative activity of Tyr-bound MB to the activity of the Tyr positive control. Regarding the red line on Panel A, the number of MBs were calculated for each condition (50, 100, 150, and 200 µL) and then used to measure dopachrome production per Tyr-MB complex. Due to a decreased probability of each MB interacting with Tyr (100 µL of 2.2 mg/mL constant for all conditions) at increasing volumes of MB, there was a decreasing trend of dopachrome formed per Tyr-MB complex (red line, Adj. R^2^ = 0.84). (**B**) features the role of temperature in the production of dopachrome by Tyr-MB complex. The number of MBs were calculated for 100 µL of MBs and divided from relative dopachrome formation (calculated as a ratio to the Tyr control as in Panel A) to show dopachrome production per Tyr-MB complex. As temperature increased, dopachrome formation per Tyr-MB complex increased (Adj. R^2^ = 0.97). (**C**) shows the relative dopachrome production normalized to the maximum 43 °C measurements for both the Tyr-MB and control and includes the 50 °C condition. The Tyr in the Tyr-MB condition is stabilized by the MB and remains functional even at the highest temperature of 50 °C. The bar graph displays a significant difference for relative dopachrome production at this temperature with the Tyr-MB condition being significantly higher than the control. When using Tyr-MB, dopachrome is still present as seen by the orange-brown color in (**D**) whereas most of the dopachrome has self-oxidized in the control condition, as seen by a clear color in Panel D. All experiments were performed in triplicate.

The binding of Tyr to MB was confirmed by using increasing volumes of MB. The test was performed by following the TBI protocol discussed in Materials and Methods using Tyr (2.2 mg/mL) for binding to the MB. 100 µL of Tyr was incubated with increasing volumes of MB that were suspended in the binding buffer (Figure 2A). A Nanodrop (A280) was used to quantify the concentration of leftover Tyr and confirmed the binding of Tyr was directly proportional to the volume of MB used. This was shown experimentally in Supplementary Figure S3 as a decrease in the concentration of Tyr remained in the supernatant when the volume of MB increased due to an increase of Tyr bound to the magnetic beads. Tyr binding is directly correlated (Adj. R^2^ = 0.78) to the volume of MB and has a Tyr binding slope of 0.0024 µg/µL^2^. 

The colorimetric activity represented by the black line in Figure 2A revealed a direct correlation (Adj. R^2^ = 0.98) of dopachrome production as the volume of MB used in each condition increased. The dopachrome formation slope of the Tyr-MB complexes was calculated to be 0.6 µM/µL.

The amount of Tyr bound per one MB particle was calculated and revealed a decrease in bound Tyr as the volume of MB increased, as shown by the red line in Figure 2A. This inverse correlation (Adj. R^2^ = 0.92) was due to a decrease in exposure of Tyr to each bead at high volumes. The volume of Tyr remained constant for each condition. Therefore, not as many molecules of Tyr were able to bind to the extended linkers (with Nickel-Nitrilotriacetic Acid, Ni-NTA) of each bead at 200 µL of MB.

Diphenol oxidase activity of Tyr was measured in units of dopachrome formation at different temperatures of 25, 31, 37, and 43 °C (Figure 2B). A 5 °C condition was used as a negative control and showed no activity of Tyr bound to MB. Figure 2B demonstrates an increase of tyrosinase activity of Tyr-MB with a growth in incubation temperature. The diphenol oxidase activity of Tyr-MB increased linearly (Adj. R^2^ = 0.97) with the temperature. The results suggest that the immobilization of Tyr to MB might improve Tyr protein stability.

This was further confirmed by incubating Tyr-MB and Tyr control under a 50 °C condition. Although most of the dopachrome self-oxidized in the control at this high temperature, the orange-brown dopachrome was preserved in the Tyr-MB condition, as seen by Figure 2D. The relative dopachrome production was also measured quantitatively and displays a significant increase of dopachrome production in the Tyr-MB condition when compared to the control at 50 °C in Figure 2C. This again validates that Tyr is stabilized in terms of remaining functional, even at higher temperatures when bound to the MB.

Dopachrome is unstable due to its auto-oxidation properties as it spontaneously loses its carboxyl group. As a result, a black and insoluble precipitate polymerizes at a pH of 7.4 as a melanin-related product. To test how the temperature affected the rate of precipitation, isolated dopachrome was placed in −80, −20, 4, or 25 °C conditions overnight. Results showed that the −80 °C condition most effectively mitigated melanin production and remained the same color before the overnight waiting period, as seen in Supplementary Figure S4.

### 2.4. Tyr-MB and Melanin: Molecular Dimensions

Atomic Force Microscopy (AFM) and Transmission Electron Microscopy (TEM) were used to characterize sizes of unbound (bare) and Tyr-bound MB. A summary of measurements performed in AFM and TEM for Tyr-MB and unbound MB are presented in Table 2 and in Supplementary Table S1, respectively.

**Figure 3:**
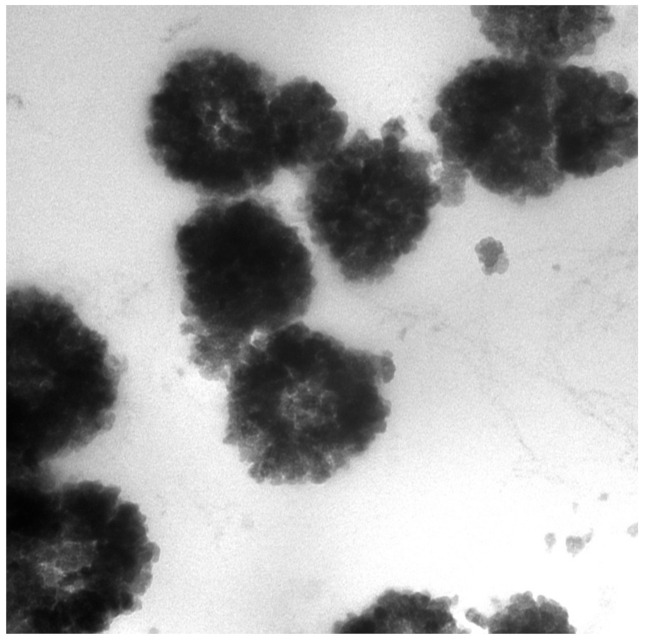
TEM images of Tyr-MB embedded into Spurr’s epoxy resin, which show the distinction between the hydrophilic outside layer and the magnetic iron oxide cluster solid core on the inside. The iron oxide cluster is displayed as light gray inside of each MB. The black color surrounding the light gray iron oxide cluster core is the hydrophilic outside layer, which is where Tyr binds. Direct magnification of 200,000×. The magnetic beads have an average diameter of 168.2 ± 24.4 nm.

TEM was performed to show images of Tyr-MB and the in vitro melanin-related product formed after overnight auto-oxidation of dopachrome. Although it was difficult to localize Tyr due to its relatively small size, TEM images showed the makeup of each MB. Bare MB consisted of an iron oxide cluster magnetic core (diameter of 49.2 ± 8.9 nm) along with an outside hydrophilic layer (MB diameter of 168.2 ± 24.4 nm). Figure 3 and Supplementary Figure S5 revealed cross-sectional slices of the MB with the relative sizes of the magnetic core and hydrophilic layer. These measurements also showed the degree to which the hydrophilic layer deformed upon attachment of Tyr. Although retaining a bumpy surface morphology, the MB was not as spherical as the bare MB once Tyr attached to the extended linkers on the hydrophilic layer.

**Figure 4:**
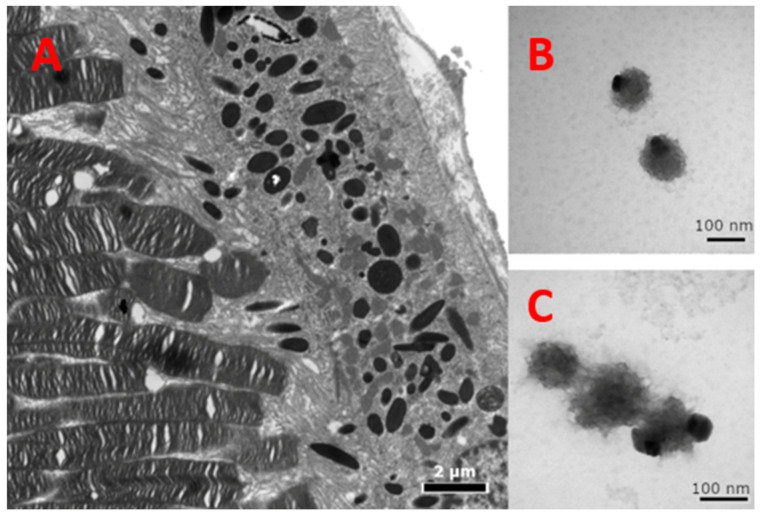
TEM of melanin-related products. (**A**) Within the RPE cells, the melanosomes are normally visualized with a dense black core of melanin, which protects the cells behind from the light. The left of the RPE are the photoreceptors, and to the right is the choroid. Direct magnification of 8000×. (**B**) Isolated spherical-shaped melanin-related beads obtained from Tyr reaction using Tyr-MB. Direct magnification of 200,000×. (**C**) A short chain of melanin-like beads assumably formed by polymerized melanin-related beads. Direct magnification of 250,000×. Less dense regions are possibly a result of inefficient polymerization due to the absence of enzymes and scaffolding present in a cell.

In Figure 4A, the dense regions of the TEM images are the melanocytes in the retinal pigment epithelium (RPE). These darker regions are comparable in color to the darker regions in the melanin-related products of Figure 4B,C. The less dense regions are due to the lack of enzymes and scaffolding that are usually present in a cell. The TEM images of the melanin-related product revealed single spherical-shaped and polymerized melanin with an average diameter of 121.4 ± 18.1 nm in Figure 4B,C.

**Figure 5:**
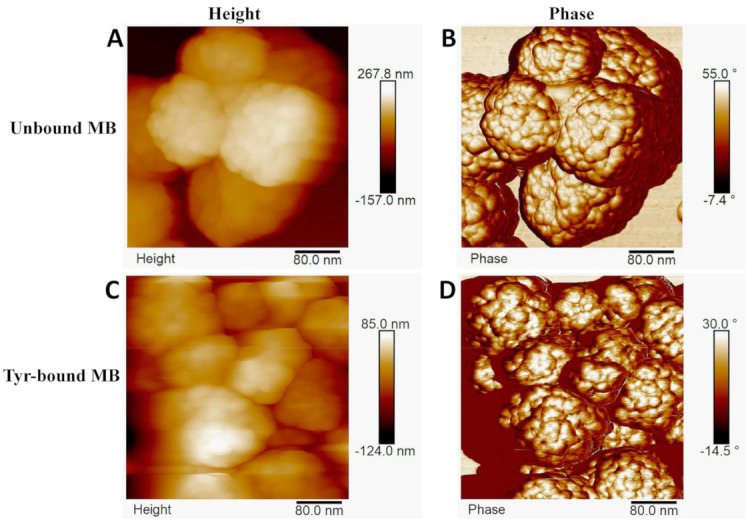
Height and phase modalities in AFM. Although the images show that Tyr-bound MB had higher aggregation sizes than bare beads due to using Poly-L-lysine-coated substrates to attach the negatively charged, hydrophilic MB particles, this was not so during experimentation. (**A**) shows the unbound MB in the height modality. (**B**) displays the unbound MB in the phase modality where you can see the fine details of the nanostructures on the hydrophilic layer of the MB, which cannot be detected in the geographical shape detection of the height modality. (**C**) displays the Tyr-bound MB in the height modality. (**D**) displays the Tyr-bound MB in the phase modality.

In AFM, the bare MB were generally neither perfectly spherical nor mono-dispersed. Their surface exhibited a rough, “bumpy” morphology with features measuring ~5–10 nm in height and ~15–20 nm at their base. Before performing AFM on Tyr-bound MB, DLS measurements revealed the hydrodynamic diameter of Tyr to be 8.30 nm, which was similar to that of measurements published previously [16]. Additionally, the isolated dopachrome products had hydrodynamic diameters of 0.87, 63.0, and 1200.0 nm. Visualization of the small size of Tyr proved to be difficult for magnetic beads coated with tyrosinase because it showed a similar bumpy (rigid) surface morphology to bare beads. Therefore, it was difficult to differentiate between surface bumps and Tyr attached to the surface of the MB (Figure 5). Although it was difficult to localize Tyr, there were differences between bare and Tyr-bound MB. Specifically, in AFM experiments, Tyr-bound MBs were observed to aggregate more strongly than bare MBs. In addition, Tyr-bound MBs attached to Poly-L-lysine-coated substrates more firmly than the bare MB particles. These observations are consistent with the expected surface charge differences between bare and Tyr-coated beads. Tyr-coated beads are expected to be strongly negatively charged at pH 7.4 (Tyr isoelectric point—pH 5.7) while the Ni^+2^ ions on the Ni-NTA linkers of the bare beads is expected to render the bead surface more positive. These observations remained the same four months later when MBs were stored at 4 °C. The surface morphology of the bare MBs, with bumps of a similar order of magnitude as the Tyr monomer, was likely the reason that the proteins themselves were not clearly visualized in the AFM height images. The phase modality of AFM can help distinguish differing surface properties, but again, in this case, the hydrophilic surface of bare MBs is likely too similar to the negatively charged and hydrophilic surface residues of the protein, making it difficult to access surface property differences between the two MBs.

## 3. Discussion

Current melanogenesis research is undermined by the absence of quantitative characterization of dopachrome-related products. Here, we propose a method to isolate dopachrome-related products from Tyr enzymatic reaction by using magnetic bead nanoparticles. Recombinant human Tyr was expressed in larvae and purified by affinity and size exclusion chromatography. The pure Tyr was immobilized to MB using 6xHis tags. L-DOPA was subjected to an oxidation reaction with the Tyr-MB nanoparticles. Dopachrome product was then isolated from the Tyr-MB particles using a magnetic separator. The catalytic activity of Tyr from Tyr-MB was measured spectrophotometrically using isolated dopachrome at 475 nm, and the similarity of tyrosinase catalytic activity was shown for intact Tyr and Tyr-MB using Hill kinetics. Furthermore, the diphenol oxidase reactions of L-DOPA were performed at different temperatures to show the increase of dopachrome formation with temperature. The molecular sizes of Tyr-MB were within limits of 168.2 ± 24.4 nm, as determined by TEM. In the AFM experiment, Tyr-MB showed some particle aggregation compared to the bare MB. In the future, Tyr-MB could be used for large-scale productions of dopachrome and melanin-related products and finding a treatment for OCA-inherited diseases.

Using metal chelate in affinity chromatography was suggested in 1975 [17]. Currently, immobilized metal-affinity chromatography (IMAC) is a separation technique that is a well-established technology for the isolation and purification of enzymes as well as proteins that are of clinical importance or used in protein biochemistry [18,19,20]. IMAC is based on the specific coordinate covalent bond of amino acids from a tag, such as a histidine tag, to metals immobilized to the chromatography resin. Further improvement in the affinity purification methods was possible after the introduction of mono-sized magnetic beads [21], which could use immobilized metal affinity for selective binding of protein molecules. At present, such magnetic beads are widely available commercially from different vendors. Here, we suggest using magnetic beads designed for IMAC to create immobilized metal-affinity nanoparticles containing an active human tyrosinase domain. Such nanoparticles could be useful for large-scale production of pure dopachrome-like products, which potentially could be implicated in clinical practice to cure the OCA disorder. This method could also have a wide range of implications for different enzymes of clinical interest.

Presented in this work, the volume-dependent, temperature-dependent, and dose-dependent experiments characterize the affinity of Tyr-MB to the L-DOPA substrate. As the volume of MB increased, so did the production of dopachrome, which confirmed that dopachrome production rose with the increasing number of Tyr-MB complexes. Additionally, higher activity of Tyr to L-DOPA substrate was detected as temperature increased (Figure 2B). According to the temperature-dependent behavior, we might expect that binding to MBs increases the protein stability of the Tyr domain, making Tyr functional at even higher temperatures (Figure 2C,D). The increased thermal protein stability in Tyr-MB showed that dopachrome of orange-brown color was produced at even high temperatures (50 °C). In contrast, for native Tyr, no color change was observed at the same temperature condition. Therefore, the native Tyr had little to no catalytic activity and had become unstable due to because of the synthesis of very little or no dopachrome. A different color of dopachrome has been obtained in optimal conditions (37 °C) for native and Tyr-MB. For the Tyr-control, the Tyr is present in solution with dopachrome formation. Therefore, the self-oxidation intensifies the production of a melanin-related product, which causes the solution to be darker and cloudier. Due to melanin-related product formation, there is a higher absorption reading at 475 nm. Finally, the dose-dependent experiment allowed us to explore the similarities between the kinetic parameters of the Tyr-MB complex and the Tyr control shown in Figure 1 with the parameters of the Hill dose-response equation calculated in Table 1. 

According to Advanced BioChemicals (https://advancedbiochemicals.com/), the molecular weight of a single MB was 284,000 ± 17,200 kDa. This was divided from the concentration of the MB stock solution (5 mg/10 µL) to calculate the number of particles in 10 µL of MB stock solution (Step 1 in Supplementary Table S2). Afterwards, the average weight of Tyr bound per MB particle was calculated by dividing the concentration of Tyr-bound MB quantified from the MB volume-dependent experiment (0.02 mg/10 µL) by the calculated number of MB particles in 10 µL of MB stock solution (Step 2 in Supplementary Table S2). Using these numbers and assuming a homogenous distribution of protein domains at the MB surface, the average number of molecules of Tyr on a single MB was calculated to be 214 ± 13 (Step 3 in Supplementary Table S2).

The tight packing of the >214 Tyr molecules on each MB would affect the catalytic activity of Tyr-MB. This would decrease the probability of L-DOPA substrate interacting with each active site of Tyr, resulting in a decreased access to Tyr, which, subsequently, lowers the production of dopachrome. During AFM, it was also observed that the MB are soft and not rigid, which suggests slight deformation upon attachment to the Tyr. This further signifies that catalytic activity of some Tyr molecules is blocked by other Tyr molecules. Therefore, Tyr could be packed more densely due to the deforming structure of the hydrophilic outer layer of the MB once Tyr molecules attach to the extended linkers.

Characterization of each MB particle through TEM (Figure 3) and AFM (Figure 5) showed that each bare bead has a spherical shape with a bumpy outer layer containing randomly allocated extended linkers that served as Ni-NTA attachment points for Tyr. AFM revealed that, upon attachment of the Tyr, the structure of the bead deformed specifically on the hydrophilic outer layer.

The shelf life of purified dopachrome was studied at different temperatures with −80 °C being the optimal condition. We found that the −80 °C condition prevented auto-oxidation of dopachrome most effectively with the least melanin production compared to −20 °C, 4 °C, and 25 °C conditions and retained its orange-brown color overnight (Supplementary Figure S4). This experiment is further discussed in the Supplementary Materials.

It was recently demonstrated that a CRISPR/CAS9-mediated mutation of tyrosinase could induce graying in rabbit [22]. From our work, the potential for OCA1A therapy could begin with direct-delivery testing of the toxicity of dopachrome in HEK cell cultures followed by an 3-(4,5-dimethylthiazol-2-yl)-2,5-diphenyl tetrazolium bromide (MTT) assay [23], or flow cytometry-based assay for apoptotic markers such as Annexin V. The toxicity of dopachrome is currently being examined in the National Eye Institute (unpublished data). Additionally, iPS-derived retinal pigment epithelium (RPE) cells [24] could be used for rescuing pigmentation defects due to OCA1A caused by the biallelic mutation of Tyr. Isolated dopachrome would be added in a dose-dependent fashion to test for pigmentation rescue.

The obtained dopachrome-like products and melanin from our method could be applied in clinical settings. For example, in photothermal therapy, melanin-like nanoparticles are developed as a treatment of human tumors and bioimaging [25,26,27]. 

Applying this method of dopachrome isolation for mutant Tyr proteins would potentially elucidate the thermodynamic parameters behind Tyr binding and assist in the search for potential activators and inhibitors in mutant variants. Furthermore, this isolation technique can be applied to other enzymes in the melanogenesis pathway like Trp 1 or Trp 2. Combining bound MB particles with Tyr, Trp 1, and Trp 2 would allow us to manipulate the melanogenesis pathway by changing the ratio of each protein in a controlled environment. Additionally, this would allow us to test if the melanin content changes with each condition. Following a reaction, the protein-bound MB complexes could also be reused. After removing the products of reaction using a magnetic separator, the protein-bound MB complexes would then be washed with 1× PBS and then used in a reaction again with consistent experimental results. This could be employed on a large-scale for mass product generation in a biochemical reactor.

In conclusion, we used magnetic beads to purify dopachrome from a mixture with Tyr protein. Traditionally, immobilized metal-affinity chromatography (IMAC) is a separation technique that is well-established technology for the isolation and purification of proteins that are of clinical or protein biochemistry importance. Here, we suggest using magnetic beads designed for IMAC to create immobilized metal-affinity nanoparticles coated with an active human tyrosinase domain. Such nanoparticles could be useful for large-scale production and characterization of pure dopachrome-like products, which potentially could be implemented in clinical practice to cure an OCA disorder. This method could have a wide range implication for different enzymes of clinical interest. We suggests a quantitative characterization of isolated dopachrome to further understand Tyr function, explore the role of OCA1-related mutations in Tyr, and characterize the products of Tyr enzymatic reaction including melanin, which is the auto-oxidized product of dopachrome. Overall, with OCA in focus, achieving dopachrome isolation and examining the thermodynamic parameters of Tyr will serve the objectives of finding a treatment for OCA-inherited diseases and elucidating the molecular mechanism of the melanogenesis pathway.

## 4. Materials and Methods

### 4.1. Tyrosinase Expression and Purification

The protein expression and purification steps were performed as previously described [9,10]. Whole *Trichoplusia ni* (*T. ni*) larvae were used for production of the recombinant truncated Tyr (native protein residues 19–469). The infected larvae were frozen at −80 °C and homogenized in 5× (*v/w*) lysis buffer (20 mM sodium phosphate, pH 7.4, 500 mM NaCl, 20 mM imidazole, 2 mM MgCl_2_, 0.2 mg/mL lysozyme from chicken egg whites (Sigma-Aldrich, Oakvil, ON, Canada), 25 µM 1-Phenyl-2-thiourea (Sigma-Aldrich, Saint Louis, MO, USA), 40 µg/mL DNAse I (Thermo Fisher Scientific, Waltham, MA, USA), and protease inhibitor tablets (Roche Diagnostics, San Francisco, CA, USA). The lysate was incubated at 25 °C for 30 min, sonicated at 25 °C for 10 min, and centrifuged at 8000 rpm at 4 °C for 30 min. The lysate was filtered and went under a 1:1 dilution *v/v* with an affinity binding buffer (20 mM sodium phosphate, pH 7.4, 500 mM NaCl, 20 mM imidazole). The C-terminal 6-His tagged recombinant truncated Tyr was isolated using a combination of IMAC and SEC. Both chromatographies were performed using an ÄKTAxpress protein purification workstation (GE Healthcare, Silver Spring, MD, USA).

A 5-mL His-Trap FF Crude IMAC column (GE Healthcare, Silver Spring, MD, USA) was loaded with the diluted lysate using an affinity-binding buffer and was followed by the elution of Tyr using the affinity elution buffer (20 mM sodium phosphate, pH 7.4, 500 mM NaCl, and 500 mM imidazole). Fractions containing Tyr were collected and dialyzed (10 K MWCO SnakeSkin Dialysis Tubing) overnight in a gel filtration buffer (50 mM Tris-HCl, and pH 7.4, 1 mM EDTA, 50 µM TCEP, 150 mM NaCl). SEC using a Sephacryl S-300 HR 26/60 column (GE Healthcare, Silver Spring, MD, USA) was used to purify the proteins further. SEC standards (Bio-Rad, Hercules, CA, USA) were used to calibrate the column (bovine thyroglobulin, bovine gamma-globulin, chicken ovalbumin, horse myoglobin, and vitamin B-12). Identification of fractions containing Tyr was confirmed by performing SDS-PAGE (Bio-Rad, Hercules, CA, USA) and Western blot analysis using anti-Tyr antibodies (T311) (Santa Cruz Biotechnology, Dallas, TX, USA). The Tyr sample was concentrated using Amicon Ultra 10,000 MWCO centrifugal filter units (Merc Millipore, Burlington, MA, USA) and the concentration was quantified using a NanoDrop 2000 UV-Vis Spectrophotometer (Thermo Scientific, Waltham, MA, USA) at A_280 nm/260 nm_.

### 4.2. Tyr-MB Particles Preparation

**Figure 6:**
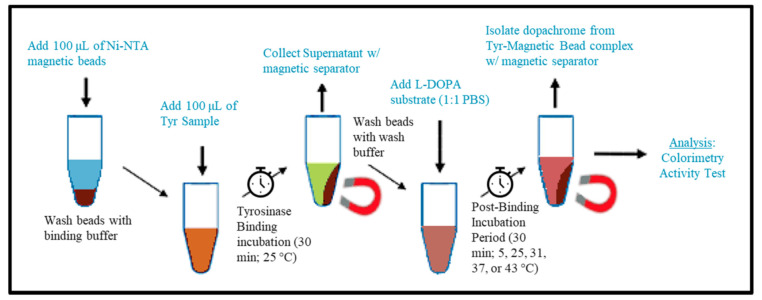
Dopachrome isolation flowchart. Dopachrome isolation begins with washing the MB with binding buffer, adding Tyr (1:1 ratio) and performing the Tyr binding incubation (TBI) for 30 min at 25 °C. The supernatant is then removed after the incubation and the beads were washed with washing buffer. Afterwards, L-DOPA is added with 1× PBS (1:1 ratio) into the tube containing MB and the final post-binding incubation (PBI) for 30 min at any temperature desired is performed. Using a magnetic separator, the dopachrome product can then be isolated for the colorimetry activity test.

The pure recombinant Tyr was immobilized to MB (Advanced Biochemicals, Lawrenceville, GA, USA), according to their protocol as follows. The procedure of Tyr-MB preparation and dopachrome isolation is shown schematically in Figure 6. In this procedure, 2 steps, known as Tyr-binding incubation and post-binding incubation for dopachrome production, were performed. The detailed protocol for each step is shown below.

### 4.3. Tyrosinase Binding Incubation (TBI) 

The experimental setup included four Eppendorf tubes for each trial. Tube 1 contained 100 µL of the MB, which were first washed (500 µL of binding buffer) and resuspended with 100 µL of the binding buffer (20 mM Tris-HCl, pH 8.0, 1 mM imidazole, 0.5 M NaCl). Tube 2 was labeled for the supernatant after the Tyr binding incubation period (TBI). Tube 3 and 4 were labeled as “wash 1” and “wash 2” and were for collection after wash buffer was added to verify Tyr was bound to the magnetic beads. Tube 5 was for the positive control (Tyr) and Tube 6 contained the negative control (1× PBS). After adding 100 µL of the binding buffer to tube 1 (containing MB), 100 µL of Tyr (1:1 ratio) was added to the suspension. Tubes 1, 5, and 6 were immediately placed on an end-to-end rotator (Benchmark Scientific, Sayreville, NJ, USA) at 25 °C for 30 min. The technique for binding Tyr to MB were repeated in triplicate and were executed independently for each temperature condition (5, 25, 31, 37, and 43 °C in the temperature-dependent experiment) or volume of MB condition (50, 100, 150, and 200 µL).

### 4.4. Post-Binding Incubation for Dopachrome Production (PBI)

After the TBI, the supernatant of tube 1 was collected in tube 2 using a magnetic separator (Qiagen, Germantown, MD, USA). The concentration of the supernatant was determined using a NanoDrop 2000 UV-Vis Spectrophotometer and confirmed that the concentration decreased consistently by a factor of 11 (2.2 mg/mL to 0.2 mg/mL Tyr) due to Tyr binding to MB. Subsequently, 1 mL of wash buffer (10 mM Tris-HCl, pH of 8.0, 5 mM imidazole, 250 mM NaCl, 0.05% Tween-20) was pipetted into tube 1 twice and the wash was collected into tubes 3 and 4 each time. The MB were washed with 1× PBS a third time afterward. The MB were then resuspended with 100 µL of 1× PBS in tube 1. This was followed by adding 100 µL of the 3 mM L-DOPA solution (1:1 ratio), as described in the colorimetric activity measurement method below, into tubes 1, 2, 5, and 6. These tubes were immediately followed with the post-binding Tyr-MB incubation in a MaxQ 4000 Orbital Shaker (Thermo Scientific, Waltham, MA, USA) for 30 min (270 rpm) at each temperature condition of 5, 25, 31, 37, and 43 °C, or MB volume condition at 50, 100, 150, and 200 µL.

### 4.5. Colorimetric Activity

Tyr diphenol oxidase activity visualized by the production of dopachrome was measured spectrophotometrically (475 nm) using a SpectraMax i3 multi-mode microplate reader detection platform (Molecular Devices, San Jose, CA, USA). A 3 mM L-DOPA (Sigma-Aldrich, Saint Louis, MO, USA) stock solution was prepared in 10 mM sodium phosphate (pH 7.4) as a substrate for Tyr [28]. 

The activity of Tyr-MB was measured alike the protocol described above. However, unlike traditional experiments, 100 µL of the L-DOPA solution was added to each Tyr-MB tube (containing Tyr-MB and 100 µL of 1× PBS), supernatant, and control to get a final 1.5 mM L-DOPA concentration (1:1 ratio). After the PBI, 100 µL of the dopachrome product (copper color indicates the reaction occurred) was collected from each Tyr-MB tube and loaded in the wells of the microplate. The supernatants of the TBI were also loaded to verify that the Tyr was bound to the magnetic beads. In addition, 100 µL of the L-DOPA solution was added to the positive control (Tyr) and the negative control (1× PBS) at a 1:1 ratio in separate wells for comparison. An endpoint reading was measured for each condition on the SpectraMax. Measurements in triplicate were independently repeated for each temperature condition in the temperature-dependence experiment or volume of the MB condition. An absorbance measurement of the isolated dopachrome at 280 nm (A_280_) using the Nanodrop suggested that there was no protein present and confirmed the purity of the dopachrome.

A 50 °C condition was also performed in triplicate for the Tyr-MB and control. Due to the high rate of self-oxidation as a result of this high temperature during the PBI, melanin-related products were produced. To separate the melanin-related products from dopachrome, both the isolated dopachrome and the control mixture were then centrifuged for 15 min using an Eppendorf Microcentrifuge 5415 R (Sigma-Aldrich, Saint Louis, MO, USA). Measurements on the SpectraMax were obtained without the pellet that contained melanin-related products.

### 4.6. Different Volumes of MB

Tyr was bound to increasing volumes (50, 100, 150, and 200 µL) of Ni-NTA MB by incubating the Tyr sample (2.2 mg/mL) with MB at room temperature for 30 min and the supernatant was removed using a magnetic separator. During the PBI, the temperature remained the same for every condition. Tyr activity for dopachrome formation was measured, according to the colorimetric activity measurement method, after the PBI at 37 °C (30 min) for every condition of volume of MB (50, 100, 150, and 200 µL). 

### 4.7. L-DOPA Dose-Dependence

The catalytic effectiveness of Tyr-MB particles is not evident compared to that of pure Tyr. We attempt to answer this question by performing a dose-dependent experiment. The experimental setup consisted of eight conditions for the MB tubes. Each came with different concentrations (12 mM, 6 mM, 3 mM, 1.5 mM, 0.75 mM, 0.375 mM, 0.1875 mM, and 0.09375 mM) of the L-DOPA substrate during the PBI. This was to analyze dose-dependence of the Tyr complexed with the MB. Additionally, there were eight conditions using the same eight different concentrations for the positive control (Tyr not complexed with MB) to compare and analyze dopachrome formation with the Tyr-MB dopachrome formation reactions after the PBI. Finally, eight conditions consisting of the same eight concentrations were used for the negative controls (1× PBS) to verify that there was no colorimetric reaction and to confirm that the L-DOPA solutions were prepared consistently. All MB tubes followed the TBI protocol and were washed with the wash buffer and, finally, the 1× PBS buffer. Serial dilutions were performed to prepare each solution of L-DOPA substrate in 10 mM sodium phosphate buffer for each of the eight concentrations. After adding the L-DOPA substrate (1:1 ratio with 1× PBS) and performing the PBI, the colorimetric activity measurement method was followed to analyze dopachrome formation. All measurements were performed in duplicate.

### 4.8. Melanin Isolation

After the PBI, dopachrome was isolated from the Tyr-MB using the magnetic separator and an endpoint colorimetric activity measurement was taken. The isolated dopachrome was then placed in −80, −20, 4, or 25 °C overnight to test for melanin production after spontaneous transformation of dopachrome. The assumed melanin-related product had a black color. The product was isolated by centrifugation for 30 min at room temperature and 13,200 rpm on an Eppendorf Microcentrifuge 5415 R for DLS, AFM, and TEM analysis. 

### 4.9. Molecular Sizes of Tyr-MB and Dopachrome Products

#### 4.9.1. Dynamic Light Scattering

DLS was performed with the Litesizer 500 Particle Analyzer (Anton-Paar USA, Ashland, VA, USA) and used to measure the hydrodynamic diameters of Tyr and dopachrome-derived products. The device has a measuring range of 0.3 nm–10 µm for particle diameter specifications. The time-dependent variations in scattered light intensity are determined due to particles encountering Brownian motion. The particle’s diffusion coefficient can be converted to size distribution by analyzing the intensity variations.

#### 4.9.2. Atomic Force Microscopy

Magnetic bead (Advance Biochemicals, 50 mg/mL) solutions were diluted by a factor of 100 or 200 and 5 µL of the dilution were deposited to either freshly cleaved mica or to mica that was coated with poly-L-lysine (Sigma-Aldrich, St. Louis, MO, USA, 30–70 kDa, P/N P2636), according to the standard protocol. The samples were incubated for 10 min and then washed with ultrapure water to remove buffer salts and dried in a stream of N_2_. These were then imaged on a commercial AFM instrument (MultiMode-8 AFM, Bruker-nano, Inc, Santa Barbara, CA, USA) using silicon cantilevers (FESP-V2 or TEST-SS, Bruker-nano, Santa Barbara, CA, USA) in the tapping mode. Images were acquired at high resolution (~1 nm/pixel) and rocessed using the instrument software (Nanoscope Analysis V.2.0).

#### 4.9.3. Transmission Electron Microscopy (TEM)

The TEM preparation was carried out according to Ogilvy et al. [29]. The preparation of the magnetic bead-bound tyrosinase was fixed in PBS-buffered glutaraldehyde (2.5%), concentrated in an Eppendorf tube, gelled in agarose (1.5% low melting point agarose), treated with PBS-buffered osmium tetroxide (0.5%), dehydrated, and embedded into Spurr’s epoxy resin. Ultra-thin sections (90 nm) were made and stained with uranyl acetate and viewed in a JEOL JEM 1010 transmission electron microscope.

Whole-mount TEM grids were prepared as follows. Melanin concentrate and dopachrome-melanin concentrate were placed in Eppendorf tubes, fixed in PBS-buffered glutaraldehyde (2.5%), and rinsed with PBS (3×). An aliquot of 5 µL from each was pipetted on a formvar carbon-coated mesh grid (Electron Microscopy Sciences, Hatfield, PA, Cat. #FCF200-Cu), air dried, stained with uranyl acetate, and viewed in a JEOL JEM 1010 transmission electron microscope.

Melanosomes in the RPE of mouse eyes were used for comparison regarding the color and density of the melanin-related product formed in vitro. Mouse eyes were inoculated and immediately fixed in 2.5% buffered glutaraldehyde, and then processed for transmission electron microscopy (TEM), according to Ogilvy et al. [29]. Specimens were washed in phosphate buffered saline (PBS), post-fixed in 0.5% osmium tetroxide (OsO4), rinsed, dehydrated, and then embedded in epoxy resin. Blocks were sectioned at about a 90-nm thickness on a Leica EM UC6 ultramicrotome (Leica, Austria), double-stained with uranyl acetate and lead citrate, and imaged with a JEOL JEM-1010 electron microscope (JEOL, Japan).

## Figures and Tables

**Table 1:** Parameters of the Hill dose-response equation for Tyr-MB compared to the values for dopachrome production in the Tyrosinase control.

	Tyr-MB	Tyr-Control
DCmax	1.20 ± 0.10	1.11 ± 0.05
DC0	0.06 ± 0.01	0.06 ± 0.01
L-DOPA50	3.18 ± 0.35	3.01 ± 0.31
n	1.65 ± 0.12	1.71 ± 0.16
Adj. R-squared Value	0.99	0.99

Note: DC, dopachrome. DCmax, maximum response in dopachrome production. L-DOPA50, L-DOPA concentration that produces a 50% of maximal response. n, Hill coefficient. Hill equation: DC/(DCmax-DC0) = 1/(1+(L-DOPA50/L-DOPA)^n^).

**Table 2:** AFM and TEM measurements on the makeup of the MB.

	Measurement Ranges (nm)
Bare Magnetic Beads (Advanced BioChemicals)	500
Tyr-Bound MB (TEM)	150–200
Tyr-Bound (AFM)	150–250
Surface “bump” diameter at base (AFM)	15–20
Surface “bump” height (AFM)	5–10

## Data Availability

The data presented in this study are openly available in IJMS at this paper.

## Supplementary Materials

The following are available online at https://www.mdpi.com/1422-0067/22/2/734/s1. Table S1: MB molecular sizes. Table S2: Calculating the number of Tyr molecules bound to each MB particle. Figure S1: Human Tyr protein purification. Figure S2: Dopachrome formation after the dopachrome isolation technique under 37 °C. Figure S3: Dopachrome production. Figure S4: Optimal dopachrome storage condition. Figure S5: TEM images of 3 different sized sections of Tyr-MB embedded into Spurr’s epoxy resin.

## Abbreviations

OCA1	oculocutaneous albinism type 1
OCA1A	oculocutaneous albinism type 1A
OCA1B	oculocutaneous albinism type 1B
Tyr	human recombinant tyrosinase
L-DOPA	l-3,4-dihydroxyphenylalanine
DHICA	5,6-Dihydroxy-1H-indole-2-carboxylic acid
MB	magnetic beads
Tyr-MB	tyrosinase immobilized to MB
TBI	Tyrosinase binding incubation period
PBI	post-binding incubation
DLS	dynamic light scattering
AFM	atomic force microscopy
TEM	transmission electron microscopy
