# Supplementary material for: Tyrosinase Nanoparticles: Understanding the Melanogenesis Pathway by Isolating the Products of Tyrosinase Enzymatic Reaction
Source: Int J Mol Sci. 2021 Jan 13;22(2):734. doi: 10.3390/ijms22020734 (PMC7828394; doi:10.3390/ijms22020734)
Supplement: Supplementary file 1 [file ijms-22-00734-s001.pdf]

Storage of Dopachrome

Dopachrome is unstable due to its auto-oxidation properties, as it spontaneously loses its carboxyl group. As a result, a black and insoluble precipitate polymerizes at a pH of 7.4 as a melanin-related product. To test how temperature affected the rate of precipitation, isolated dopachrome was placed in −80, −20, 4, or 25 ^{o}C conditions overnight. Results showed that the −80 ^{o}C condition most effectively mitigated melanin production and remained the same color as that before the overnight waiting period, as seen in Supplementary Figure S4. Obtaining an ideal dopachrome storage temperature is necessary because of the need to minimize auto-oxidation of dopachrome for application to future experiments. For example, it is important for testing toxicity of dopachrome in HEK cell cultures where avoiding auto-oxidation would help collect more accurate data when followed by an MTT assay or flow cytometry-based assay to check for apoptotic markers. It would also be necessary when testing to see if the addition of dopachrome to iPS-derived retinal pigment epithelium (RPE) cells can account for rescuing pigmentation defects due to OCA1A in the RPE.

Supplementary Tables

Supplementary Table S1. MB molecular sizes.

	**Whole MB-magnetic core and hydrophilic outside layer (nm)**	**Iron oxide cluster magnetic core (nm)**
TEM diameter measurement	168.2 ± 24.4	49.2 ± 8.9

Supplementary Table S2. Calculating the number of Tyr molecules bound to each MB particle.

	**Steps:**	**Calculating How Many Tyr Molecules Are Attached to Each MB Particle**
	Concentration of MB stock solution	0.50 mg/ 10 μL
	Estimated Molecular Weight of single MB particle via Marvin Particle Size Analyzer (mg)	4.72E-13 ± 2.86E-14
Step 1:	Number of MB particles in 10 μL	1.06E + 12
	Weight of Tyr bound per 10 μL (mg)	0.02 mg/10 μL
Step 2:	Weight of Tyr bound per MB particle (mg)	1.89E-14
	Weight of single Tyr molecule (mg)	8.82E-17
Step 3:	Number of Tyr molecules in each MB particle	214.12

Supplementary Figures

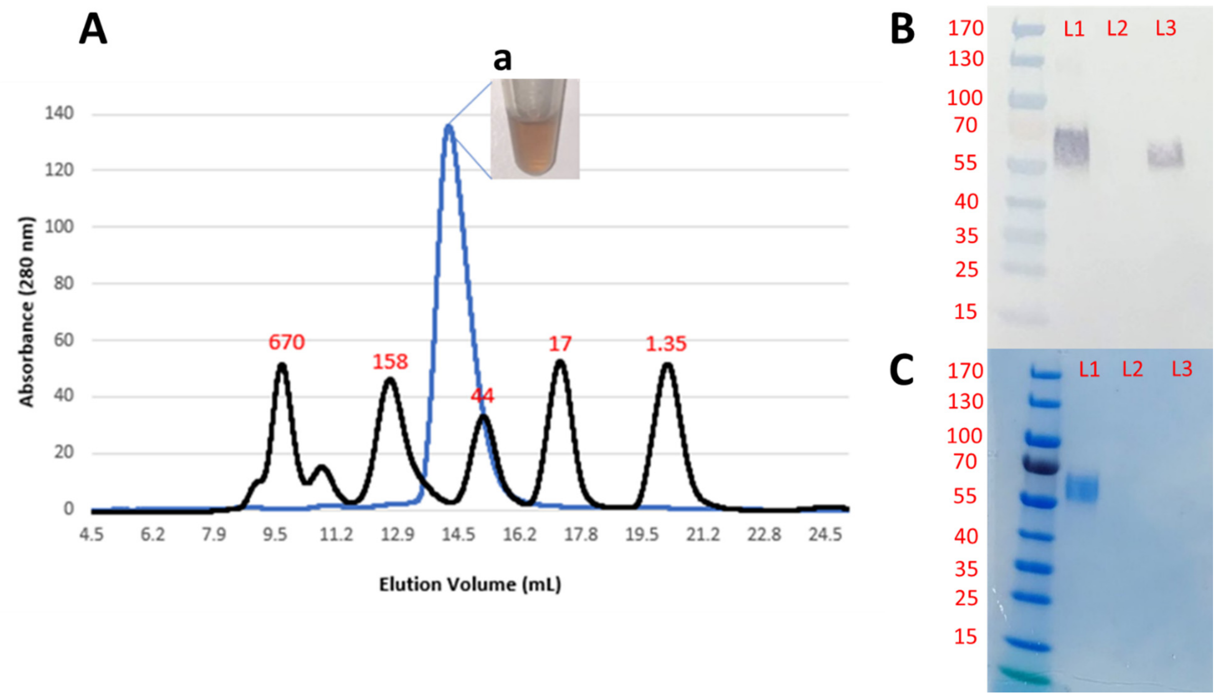

Supplementary Figure S1. Human Tyr protein purification. (**A**), A single chromatography peak for catalytically active human recombinant Tyr was obtained after one step of IMAC and two steps of SEC (16/600 Superdex 200 pg followed by Superdex 200 Increase) using the ÄKTA pure chromatography. The blue peak is the purified Tyr and the black peaks are the protein standards. The standards from left to right are thyroglobulin, gamma globulin, ovalbumin, myoglobin, and vitamin B-12, respectively. The molecular weight in kDa is displayed in red. The activity of the pure Tyr was confirmed with the activity test by adding L-DOPA at a 1:1 ratio. A brown dopachrome (Insert **a**) is formed after the Tyr reaction and is shown next to the blue peak. Western blot (**B**) and SDS-PAGE (**C**) before and after using MB. Lanes L1, L2, and

L3 correspond with the Tyr before the TBI period with MB, the supernatant after the TBI period, and the elution of the Tyr from the MB using the imidazole buffer, respectively. Although, the Western blot confirms that Tyr was present after eluting the Tyr from the MB. Due to its low concentration, it is not seen in the gel.

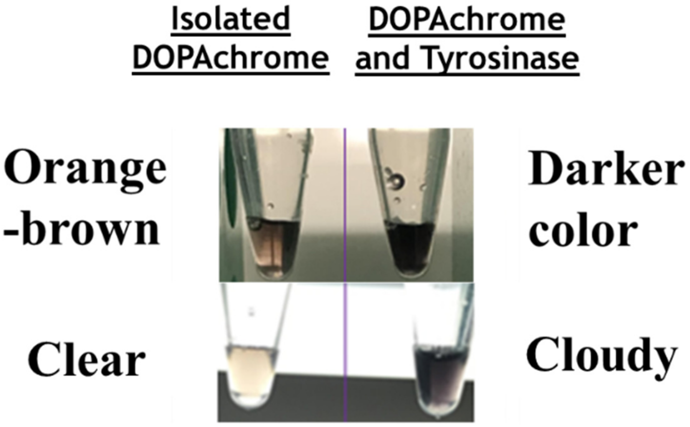

Supplementary Figure S2. Dopachrome formation after the dopachrome isolation technique under 37 °C. After isolating dopachrome from Tyr using MB, an orange-brown colored dopachrome had formed in PBS. Due to the presence of Tyr in a mixture with dopachrome formation within the Tyr-control condition, there is higher self-oxidation, leading to the production of a melanin-related product. The product causes the solution to be darker and cloudier in comparison to the isolated orange-brown dopachrome obtained using magnetic beads. Due to melanin-related products being formed in the Tyr-control condition, there is a higher absorption at 475 nm.

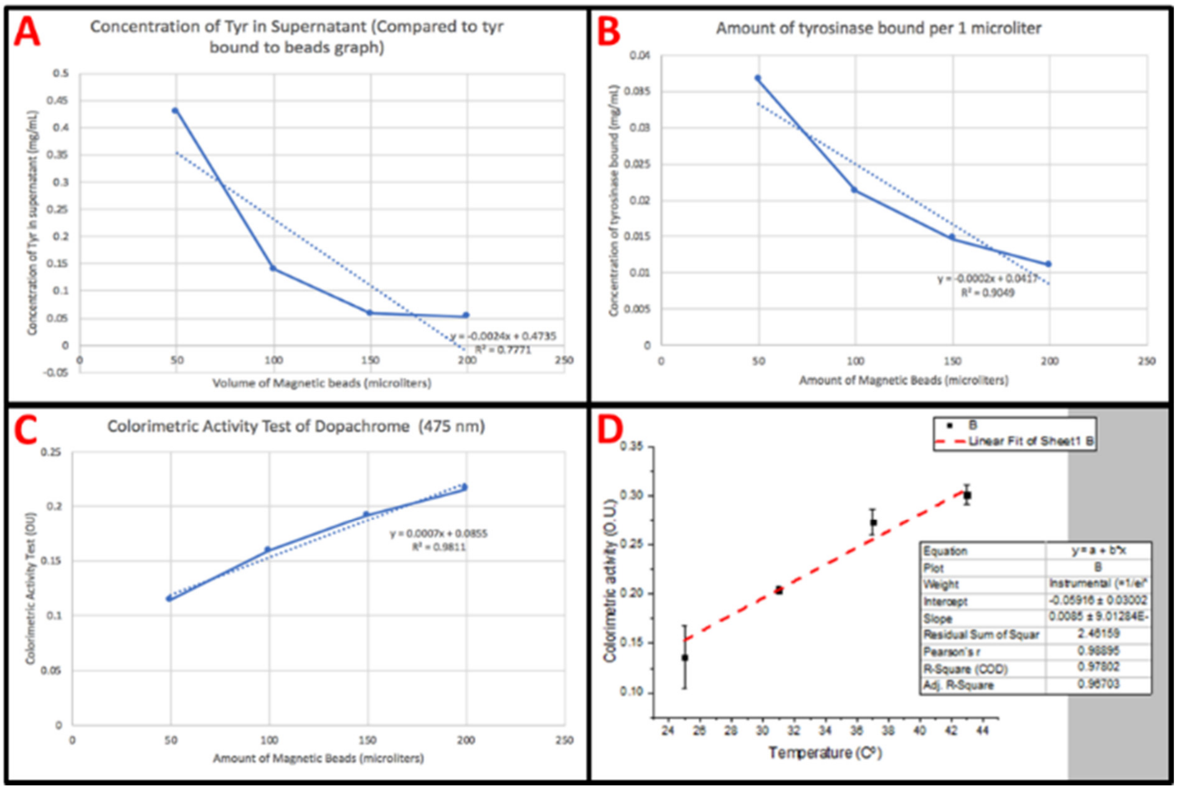

Supplementary Figure S3: Dopachrome production. (**A**) Concentration of Tyr in supernatant. (**B**) Concentration of Tyr bound to each microliter of magnetic beads. (**C**) Colorimetric test for each condition of magnetic bead volume at 50, 100, 150, and 200 μL. (**D**) Temperature-dependent experiment used to test the activity of Tyr-MB in different temperature conditions (25, 31, 37, and 43 °C).

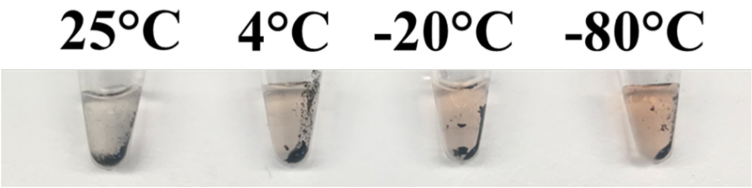

Supplementary Figure S4: Optimal dopachrome storage condition. Different temperatures were tested (25, 4, −20, and −80 °C) to find the ideal storage condition that produced the least amount of melanin. Minimizing the auto-oxidation of dopachrome for future experiments (detailed in the discussion above) is crucial with respect to gathering accurate data. The lowest temperature (−80 °C) was found to minimize auto-oxidation most effectively. This is seen by the fact that this condition remained the same brown color for 24 h.

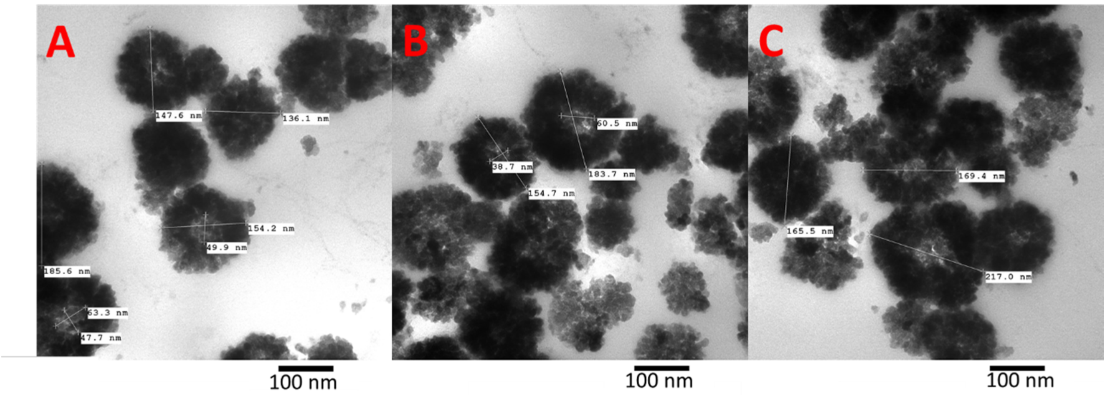

Supplementary Figure S5: TEM images of three differently sized sections of Tyr-MB embedded into Spurr's epoxy resin. Included are the measurements for diameter of both the magnetic solid core consisting of iron oxide clusters and the entire bead (including the hydrophilic outside layer). Direct magnification of 200,000x. **Panel A:** Show magnetic beads with an average diameter of 162.87 nm. **Panel B:** Show magnetic beads with an average diameter of 169.2 nm. **Panel C:** Features magnetic beads with an average diameter of 183.97 nm.
